# Agricultural landscapes and the Loire River influence the genetic structure of the marbled newt in Western France

**DOI:** 10.1038/s41598-018-32514-y

**Published:** 2018-09-21

**Authors:** Jean-Marc Costanzi, Pascal Mège, Alexandre Boissinot, Francis Isselin-Nondedeu, Sandra Guérin, Olivier Lourdais, Audrey Trochet, Quentin Le Petitcorps, Agathe Legrand, François Varenne, Pierre Grillet, Sophie Morin-Pinaud, Damien Picard

**Affiliations:** 1 Faculty of Technology, Natural Sciences and Maritime Sciences, University of South-Eastern Norway, Gullbringvegen 36, Bø i Telemark, 3800 Norway; 2 Département de Biologie, UFR de Sciences, Université d’Angers, 2 Boulevard de Lavoisier, 49000 Angers, France; 3 Centre d’Études Biologiques de Chizé, CNRS et Université de la Rochelle – UMR 7372, F-79360 Villiers en Bois, France; 4 Ecole Polytechnique de l’Université François Rabelais, Département d’Aménagement et d’Environnement, UMR 7324 – CNRS CITERES 33-35 allée Ferdinand de Lesseps, 37200 Tours, France; 5 IMBE, UMR Université Aix-Marseille Avignon, 7223-CNRS, 237-IRD IRPNC (Ingénierie de la Restauration des Patrimoines Naturels et Culturels), Avignon, France; 6 EPHE, PSL Research University, CNRS, UM, SupAgro, IRD, INRA, UMR 5175 CEFE, F-34293 Montpellier, France; 7 CNRS, ENFA, UMR5174 EDB (Laboratoire Evolution et Diversité Biologique), Université Paul Sabatier, 118 route de Narbonne, Toulouse, F-31062 France; 8 Station d’Ecologie Théorique et Expérimentale, UMR 5321, Moulis, F-09200 France; 9 LPO Vendée - La Brétinière, 85000 La Roche-sur-Yon, France; 10 10 rue de la Sayette, 79340 Vasles, France; 11 Office National de la Chasse et de la Faune Sauvage - Unité Avifaune migratrice, Direction de la Recherche et de l’Expertise, F-79360 Villiers en Bois, France

## Abstract

Amphibians are particularly sensitive to landscape fragmentation. Potential barriers between breeding sites can negatively influence the dispersal of individuals and increase genetic structure between populations. In this study, we genotyped 10 microsatellites for 334 marbled newts (*Triturus marmoratus*) at 11 different locations in Western France. Samples were collected in different regions with contrasting agricultural landscapes (low and high proportion of arable land in the north and south, respectively). We found a strong genetic structure between the northern and southern sampling sites. Isolation by distance was recorded after 62 km, but within the northern region, little or no genetic structure was detected over large distances (up to 114 km). Genetic structure at shorter distance (43 km) was found between sites situated in landscapes with larger amounts of arable lands. A significant positive relationship was found between the pairwise genetic distance (F_st_) between sites and the amount of arable land together with the distance between sites. Our results suggest that the Loire River might act as a corridor for the marbled newt, while arable land might act as a barrier. Finally, although a large city is located between sampling sites, no effect was detected on population structure.

## Introduction

Amphibians are one of the most threatened taxa in the world, with 32.5% of the species ranked as vulnerable^1^. Among the principal causes of decline, habitat loss and fragmentation play an important role^2^. The latter can be defined as the reduction of overall available habitat, leading to the division of a favourable habitat into smaller patches surrounded by a matrix of non-favourable habitat^2–4^. Habitat fragmentation can be caused by various factors such as urbanisation, transport infrastructures, deforestation and agricultural activities^5^. It is well recognised that habitat fragmentation negatively affects populations, both at a demographic and a genetic level. For instance, decreasing habitat size has a direct, adverse effect on population size^6^. Fragmentation also results in lower migration rates between habitat patches^7^. In an isolated population, the low immigration rate of new individuals from other populations can lead to insufficient gene flow to maintain genetic diversity. This happens when isolation between populations is so strong that inbreeding and genetic drift are no longer counterbalanced by gene flow^8^. The resulting erosion of genetic diversity will negatively affect fitness and increase extinction risk^9^. It is therefore important to detect the most isolated populations so as to increase gene flow between populations and to preserve corridors.

Many amphibian species are particularly sensitive to habitat fragmentation because during their life cycle individuals must move between different habitat types. Amphibians can undergo two types of movements: migration and dispersal^10^. Migrations are the movements within the life cycle of an individual (e.g.: movement from wintering sites to reproduction sites) that happen annually, whereas dispersal involves one-way movements from one habitat patch to another. Because dispersal plays an important part in reducing genetic structure between populations, changes in landscape characteristics that greatly influence the dispersal of individuals can have strong negative impacts on populations^2^.

In France, the agricultural landscape has changed significantly since the 1950’s. After the Second World War, the growing demand for resources induced a shift from small-scale to large-scale agricultural exploitations that has led to land use modification^11^. Related agricultural practices such as tillage, drainage of wetland areas, intense grazing and use of large quantities of pesticides have negatively impacted biodiversity by transforming the landscape and making it unsuitable for certain species^12^. Moreover, the need for high productivity resulted in the transformation of small fields into large open fields at the expense of hedgerows^11^. Because some animals use hedgerows as habitat and corridors^13,14^, their destruction induced significant habitat loss and fragmentation for many species^13^.

The destruction of hedgerows in favour of large fields^11^ could negatively impact our study species, the marbled newt (*Triturus marmoratus*), which is mostly found in agricultural landscapes composed of conserved meadows, hedgerows^15^, and forest areas^16,17^. Its distribution area includes western France and the northern Iberian peninsula^18^. Despite being categorised as Least Concern by the IUCN, it is protected by Annex IV of the EU Habitats Directive and Annex III of the Bern Convention. The species is also locally endangered in central France^19^. The major threats for the marbled newt are degradation of its habitat (due to agricultural intensification, among other things), disease, non-native predators, and illegal capture for the pet trade. Like many amphibian species, marbled newt populations are declining^19^ and little is known about their population structure. Understanding the influence of landscape on the genetic structure of the marbled newt is important for taking conservation measures to restore gene flow and protect sensitive populations^8^.

Previous studies on newts reported different results at different scales regarding population structure and dispersal capacities of the marbled newt, or closely related species. Jehle *et al*.^20^ found genetic structure for the marbled newt and the crested newt (*Triturus cristatus*) at a local scale (less than 10 km) whereas Prunier *et al*.^21^ found no genetic structure at larger scale (more than 10 km) for another large newt, the alpine newt (*Ichthyosaura alpestris*). An important dispersal ability was also found for a small-bodied species, *Lissotriton helveticus*, that could rapidly colonise restored ponds in a forested landscape, resulting in a weak genetic structure^22^.

Polymorphic genetic markers, such as microsatellites, are particularly useful in observing the influence of landscape characteristics on population structure. They allow us to study the relationships between different sites and estimate the connectivity between populations at different geographical scales^2^. Depending on the studied species, the time needed to observe a change in population structure between different sites may vary. In our case, the landscape changed 70 years ago. Based on the results of Prunier *et al*.^21^ on a closely related species, the alpine newt, we estimate that this is enough time to detect the influence of agricultural landscape on population structure.

In the light of the previously cited results, we defined two main goals for this study: (1) to investigate the population structure of the marbled newt in the west of France at different scales (local, regional and inter-regional), and (2) to test the influence of the agricultural landscape on population structure. Specifically, we expected the spatial genetic structure would be mostly driven by isolation by distance, but also to some extent by landscape structure^21,23^. We also expected that the agricultural landscape would negatively influence the dispersal of the marbled newt, resulting in a higher population structure.

## Results

### Genetic diversity

Among the 10 selected loci, no significant linkage disequilibrium was found after Bonferonni correction (see Costanzi *et al*.^24^). No potential null alleles were detected, and in total the data set had 4.75% missing data.

In one sampling site (NE1), a significant deviation from the Hardy-Weinberg equilibrium was found after Bonferroni correction for four loci (Tmar 2, Tmar 17, Tmar 20, Tmar 21). No positive correlation was found between F_is_ and F_st_ over all loci of sampling site NE1. It seems that the deviation from the Hardy-Weinberg equilibrium is not due to a Whalund effect and therefore sampling site NE1 was kept for the rest of the analysis^25^.

**Figure 1:**
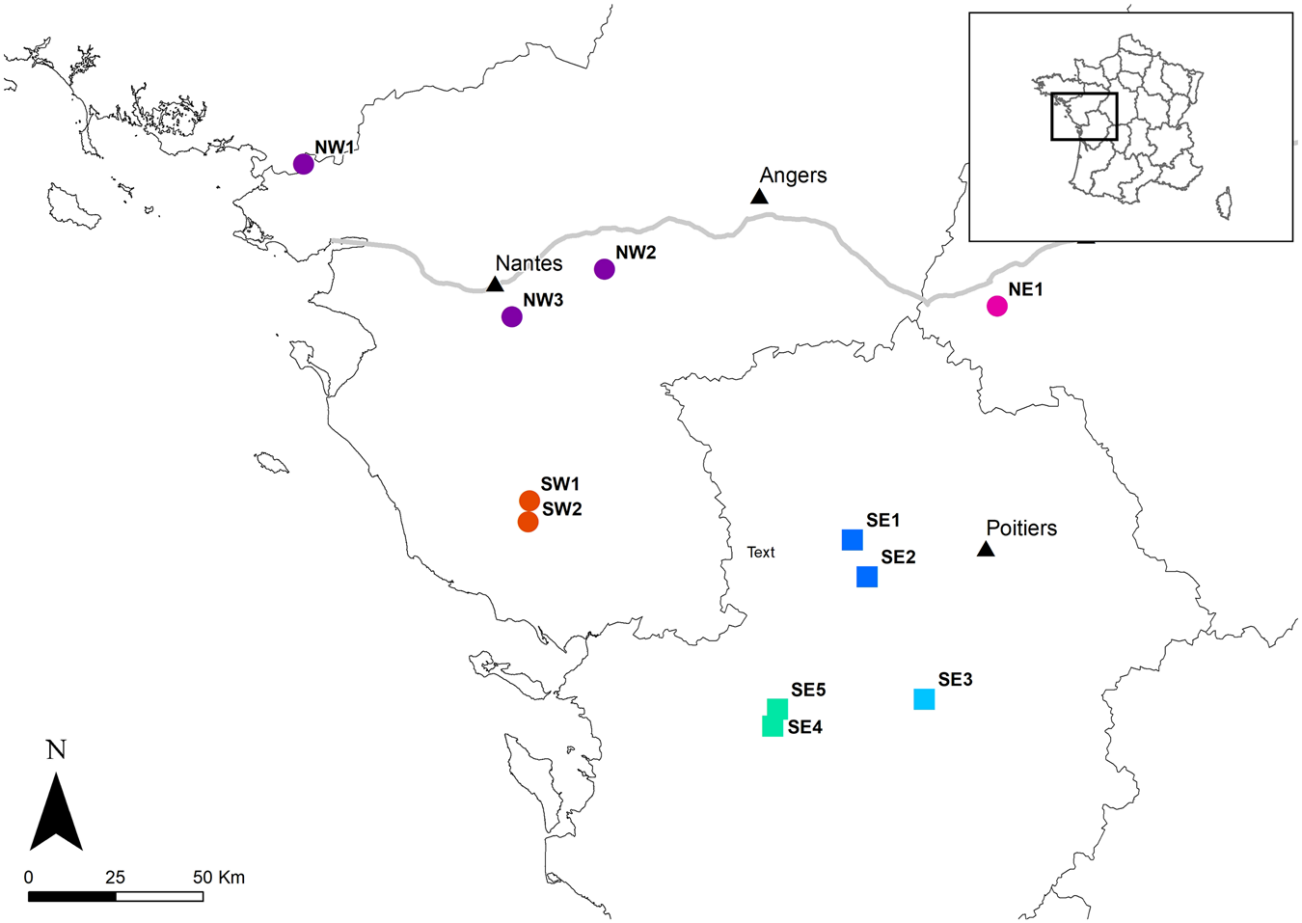
Location of the 11 sampling sites (SE1 to SE5, SW1, SW2, NW1, NW2, NW3 and NE1), the square and dots are the locations of the sites, for southern group and the northern group respectively (groups were assigned with the software STRUCTURE), and each colour represents a different cluster as defined by the software STRUCTURE. The black triangles show the major cities in the area, the black lines represent regions borders and the grey line is the Loire River. The figure was created using ArcMap 10.4 (Esri, Redlands, CA, USA, http://www.esri.com/arcgis/about-arcgis).

Among all 334 individuals distributed on 11 sites (Fig. 1), the number of alleles per locus ranged from 3 (Tmar 23) to 16 (Tmar 20). Using the rarefaction curves obtained with the R package PopGenkit (PopGenKit v1.0 R package, R Core Team), the average expected number of alleles over all loci per population for 14 individuals varied from 2.59 for NW1 to 4.45 for SE2 (Table 1).

**Table 1:** Summary statistics of genetic diversity for each locality (sampling sites): sample size, average number of alleles per locus (Avg. nb. of allels/loc), allelic richness (Ar.) (for n = 15), expected (H_exp._) and observed (H_obs._) heterozygosity.

Locality	Sample size	Avg. nb. of allels/loc	Ar.	H_exp._	H_obs._
NW1	16	2.9	2.588	0.3585	0.2672
NW3	22	3.5	3.025	0.3605	0.3297
NW2	23	4.1	3.663	0.3877	0.3819
SW1	28	4.1	3.453	0.3848	0.3611
SW2	17	4.4	4.099	0.4677	0.4388
NE1	90	5.4	3.279	0.4158	0.3387
SE2	28	5.2	4.454	0.5355	0.5097
SE1	16	4.3	4.223	0.5021	0.5313
SE3	39	4.4	3.776	0.5281	0.4793
SE5	35	4.9	4.074	0.4958	0.4552
SE4	20	4.6	4.170	0.4811	0.5050

### Genetic structure

**Figure 2:**
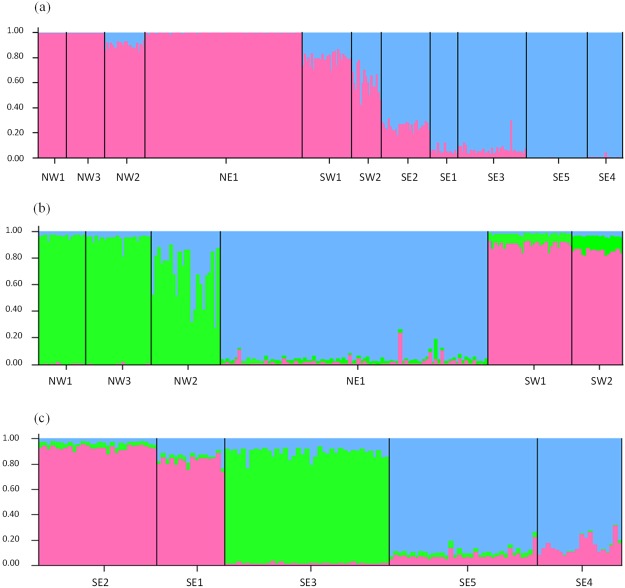
Bar plot presenting the results from STRUCTURE for the most likely the number of clusters (K) calculated with the Evanno *et al*.^66^ method. (**a**) Population structure results for all sampling sites, K = 2. (**b**) Substructure results for the northern and southwestern sampling sites (NW1, NW3, NW2, NE1, SW1, SW2), K = 3. (**c**) Substructure results for the southeastern sampling sites (SE2, SE1, SE3, SE5, SE4), K = 3.

When analysing the whole dataset with STRUCTURE^26^, the software found that a division into 2 clusters best represented the data (ΔK2 = 131.82). While most of the sampling sites were assigned to a group with an average membership coefficient >0.9, three sampling sites (SW1, SW2 and SE2) presented more admixture than the other sites with an average membership coefficient of 0.80, 0.61 and 0.74 for SW1, SW2 and SE2, respectively. Apart from these three sites a clear separation was revealed by STRUCTURE between the northern sites (NW1, NW2, NW3 and NE1) and most of the southeastern sites (SE1, SE3, SE4 and SE5) (Fig. 2a). Within each cluster, additional levels of substructure were found. For the northern and southwestern sites, three genetic clusters were detected by STRUCTURE (ΔK3 = 58.31). The first cluster grouped the southwestern sites SW1 and SW2, the second gathered the northwestern sites (NW1, NW2, NW3) and the last contained the northeastern site NE1 (Fig. 2b). No substructure was detected within these three genetic clusters when analysed separately. For southeastern sites, three clusters were detected (ΔK3 = 7.72). Sampling sites SE1 and SE2 formed the first sub-cluster, SE3 the second and finally SE4 and SE5 formed the third (Fig. 2c). No clear substructure was detected within these clusters when analysed separately.

**Figure 3:**
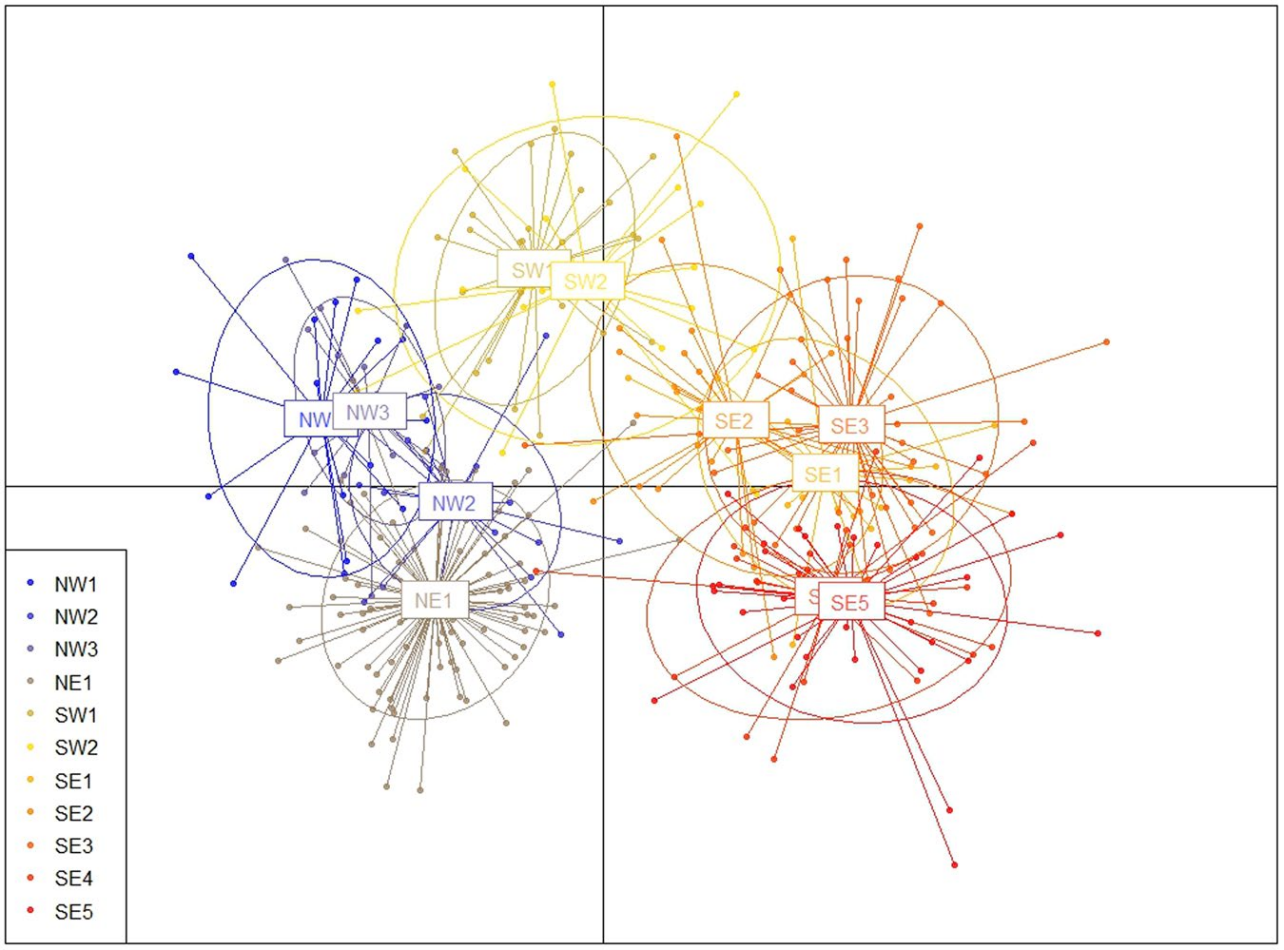
Scatterplots of DAPC. Dots are individuals, inertia ellipses and colours represent the different groups.

The results from the DAPC scatter plot presented some similarities with the results from STRUCTURE. There was a clear genetic structure between three clusters. The first cluster gathered four sites (NW1, NW2, NW3 and NE1), the second gathered the sampling sites SW1 and SW2 while the last gathered sites SE1, SE2, SE3, SE4 and SE5 (Fig. 3). A second level of substructure could be observed for the first cluster with NW1 and NW3 being genetically very close, while NE1 and NW2 seem to show some differences (with NE1 being the most different). Regarding the southeast region (SE1, SE2, SE3, SE4 and SE5), the DAPC results mostly show a division in two groups: SE1, SE2 and SE3 for the first group, and SE4 and SE5 for the second group.

After Bonferonni correction, F_st_ analysis showed a significant differentiation between most populations. No significant differentiation was only found in three instances: between sample sites NE1 and NW2 (F_st_ = 0.03587, p-value > 0.001), SE2 and SE4 (F_st_ = 0.02695, p-value > 0.001), and SE4 and SE5 (F_st_ = −0.0016, p-value = 0.55) (Table 2).

**Table 2:** Pairwise F_st_ for sampling sites (italicised F_st_ are not significantly different from 0 after Bonferonni correction). Sites with less than 20 individuals where removed from the analysis.

	NW3	NW2	NE1	SW1	SE2	SE3	SE5	SE4
NW3	0							
NW2	0.08059	0						
NE1	0.07554	*0.03587*	0					
SW1	0.18081	0.11545	0.14204	0				
SE2	0.21075	0.1303	0.1563	0.05191	0			
SE3	0.28519	0.21447	0.24568	0.17396	0.05587	0		
SE5	0.292	0.18324	0.22402	0.1305	0.03333	0.04702	0	
SE4	0.31895	0.20199	0.24632	0.14907	*0.02695*	0.05486	*−0.0016*	0

Supporting the results of STRUCTURE and DAPC analysis, the pairwise F_st_ inside the northern group were weak and less than 0.1. The same goes for the pairwise F_st_ inside the southeastern group, with weak F_st_ lower than 0.1. The results of STRUCTURE and DAPC show similar patterns as pairwise F_st_ between the northern group and the southeastern group, with strong F_st_ always higher than 0.2 (Table 2).

The STRUCTURE analysis revealed that the first cluster associated the northern group more with the southwestern group (SW1 and SW2). However, pairwise F_st_ results indicate that the southwestern sites present similar genetic distance with the southeastern sites (pairwise F_st_ for SE2, SE3, SE4, SE5 when compared to SW1, respectively: 0.0519, 0.174, 0.149, 0.131) and with the northern sites (pairwise F_st_ for NE1, NW2, NW3 when compared to SW1, respectively: 0.142, 0.116 and 0.181) (Table 2). For the DAPC results, the southwestern sites have a genetic identity in-between the northern sites and the southeastern sites. Sampling site NW2 is clearly associated with the other northwestern sites (NW1 and NW3) in the STRUCTURE analysis, while with the pairwise F_st_ it is genetically closer to the northeastern site (NE1) (F_st_ = 0.03587, p-value < 0.001). With the DAPC analysis, NW2 has an intermediate genetic identity between the other northwestern sites (NW1 and NW3) and site NE1 (northeastern site).

Regarding the Southern cluster, we detected a population structure at a smaller spatial scale than for the northern cluster. STRUCTURE and DAPC found some genetic structure between SE3 and SE4 + SE5 even though those sites are 43 km apart.

Groups for the analysis of molecular variance (AMOVA) calculation were defined according to the STRUCTURE results by creating six different groups: NW2 and NW3; SW1; NE1; SE2; SE3; SE4 and SE5. The results indicate that 84.01% of the genetic variance can be found within sampling sites, 2.47% among sampling sites but within groups and the remaining 13.52% is attributable to among-groups variability.

**Figure 4:**
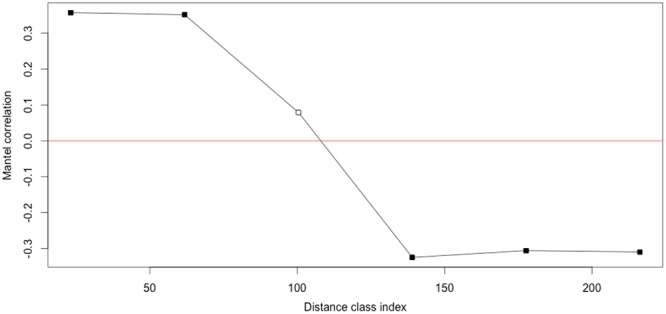
Mantel correlogram between pairwise standardised genetic distances (F_st_/1 − F_st_) and geographic distance (in km). For black squares p < 0.05, and for white squares p > 0.05.

The Mantel correlogram showed a significant positive correlation between genetic and geographic distance for the two first classes, 0 to 23 km and 23 to 62 km (p-value: < 0.01), indicating that populations separated by less than 62 km tend to be genetically similar. No significant correlation was detected for class 62 to 139 km. A significant negative correlation was detected for the two last classes, 139 to 177 km and 177 to 216 km (p-value: < 0.05) (Fig. 4).

### Influence of agricultural landscape on pairwise F_st_

**Figure 5:**
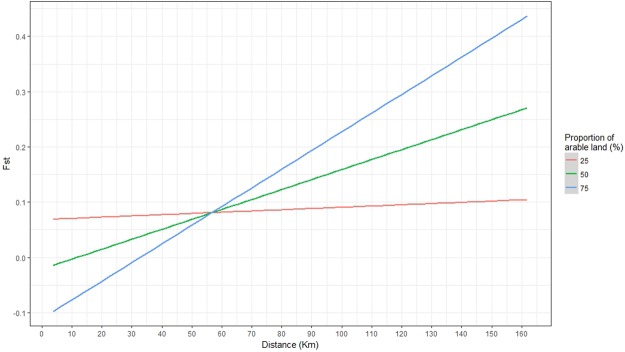
Predicted relationship of pairwise F_st_ and distance (in km) depending on different proportions of arable land between sites within a 10 km wide corridor (N = 28). The red, green and blue linear regression are for, respectively, 25%, 50% and 75% of arable land.

Pairwise F_st_ was best explained by the model including the interaction: “amount of arable land” with “pairwise distance”. The 95% confidence interval did not overlap 0 for the interaction (LCI: 2.12968e-05, UCI: 0.00011), and was therefore considered informative^27^. The effect of the interaction (Fig. 5) shows that pairwise F_st_ increased significantly with distance and with the proportion of arable land between sites.

## Discussion

Our study provides insight on the marbled newt population genetic structure at local, inter-regional, and regional scales. Most notably, we found two levels of population structure. Firstly, a strong inter-regional genetic structure between southern and northern sampling sites, and secondly regional genetic structure within those groups. No genetic structure was found at a local scale (<15 km). Moreover, isolation by distance was detected for distance greater than 62 km.

Observed and expected heterozygosity are similar to those found by Jehle *et al*.^23^, also in Western France, for the marbled newt. For the expected heterozygosity, Jehle *et al*.^23^ found a minimum and maximum of 0.20 and 0.55, respectively, while we found a minimum and maximum of 0.36 and 0.54, respectively. For the observed heterozygosity, the minimum and maximum were respectively 0.15 and 0.47 for Jehle *et al*.^23^ and 0.27, 0.53 for our study. These results indicate a similar genetic diversity between the two studies.

At inter-regional scale, over all sampling sites, the STRUCTURE results showed that “two clusters” (ΔK2 = 131.82) was the most likely scenario (Fig. 2a). However, some sampling sites (SW1, SW2 and SE2) presented more admixture than the remaining sites and were assigned to a specific cluster with a membership coefficient <0.9. It is possible that the strong difference between the northern group (NW1, NW2, NW3 and NE1) and most sites of the southeastern group (SE1, SE3, SE4, SE5) might have influenced the STRUCTURE results indicating that two clusters is the most likely scenario, while in fact sites SW1 and SW2 are in a genetically and geographically intermediate position and could therefore represent a third cluster. This is also supported by the DAPC results that displayed three distinct genetic groups (Fig. 3) over all sampling sites: the northern group (NW1, NW2, NW3 and NE1), the southwestern group (SW1 and SW2) and finally the southeastern group (SE1, SE2, SE3, SE4 and SE5). In general, the genetic structure seems to follow the geographic position of the sampling sites. We also notice that site SE2 presents some admixture in the STRUCTURE plot (Fig. 2a), and shares similarities with site SW2 in the DAPC plot (Fig. 3). In light of these results, we speculate that occasional gene flow may have occurred between the three different clusters.

Pairwise F_st_ between northern and southeastern sampling sites ranged from 0.130 (between NW2 and SE2) to 0.319 (between NW3 and SE4) (Table 2). As these regions are far apart (minimum distance is 79 km between NE1 and SE1, and maximum distance is 236 km between NW1 and SE3), isolation by distance is likely playing a role in the observed structure, as sampling sites separated by more than 62 km tend to be genetically different (Fig. 4). SW1 and SW2 might also be differentiated from the other sampling sites due to isolation by distance, as they are separated by more than 62 km from most of the sites (only NW3 is separated by 53 and 59 km from SW1 and SW2, respectively). This pattern of isolation by distance is relatively high when compared to the results of Prunier *et al*.^21^ for the alpine newt. In their study, Prunier *et al*.^21^ found evidence of isolation by distance for samples 12 km apart or more. However, our results seem to be closer to those of Emaresi *et al*.^28^ who found no isolation by distance pattern over their study area with sampling sites separated by a maximum of 26 km. Our results of isolation by distance suggest some long distance connectivity, which according to Kimura and Weiss^29^ could indicate that gene flow between sites follows a stepping stone model.

A second set of analyses on each of the previous clusters originally identified by the software STRUCTURE yielded a second level of intra-regional structure, distinguishing 6 different groups (Fig. 2b,c). The AMOVA results were in accordance with the groups defined by STRUCTURE. Indeed, the “among populations within group” variation was very low (2.47%), meaning that individuals from the same group have similar alleles. There was a greater difference between groups (13.52%) corroborating the results found with STRUCTURE and F_st_ calculation.

**Figure 6:**
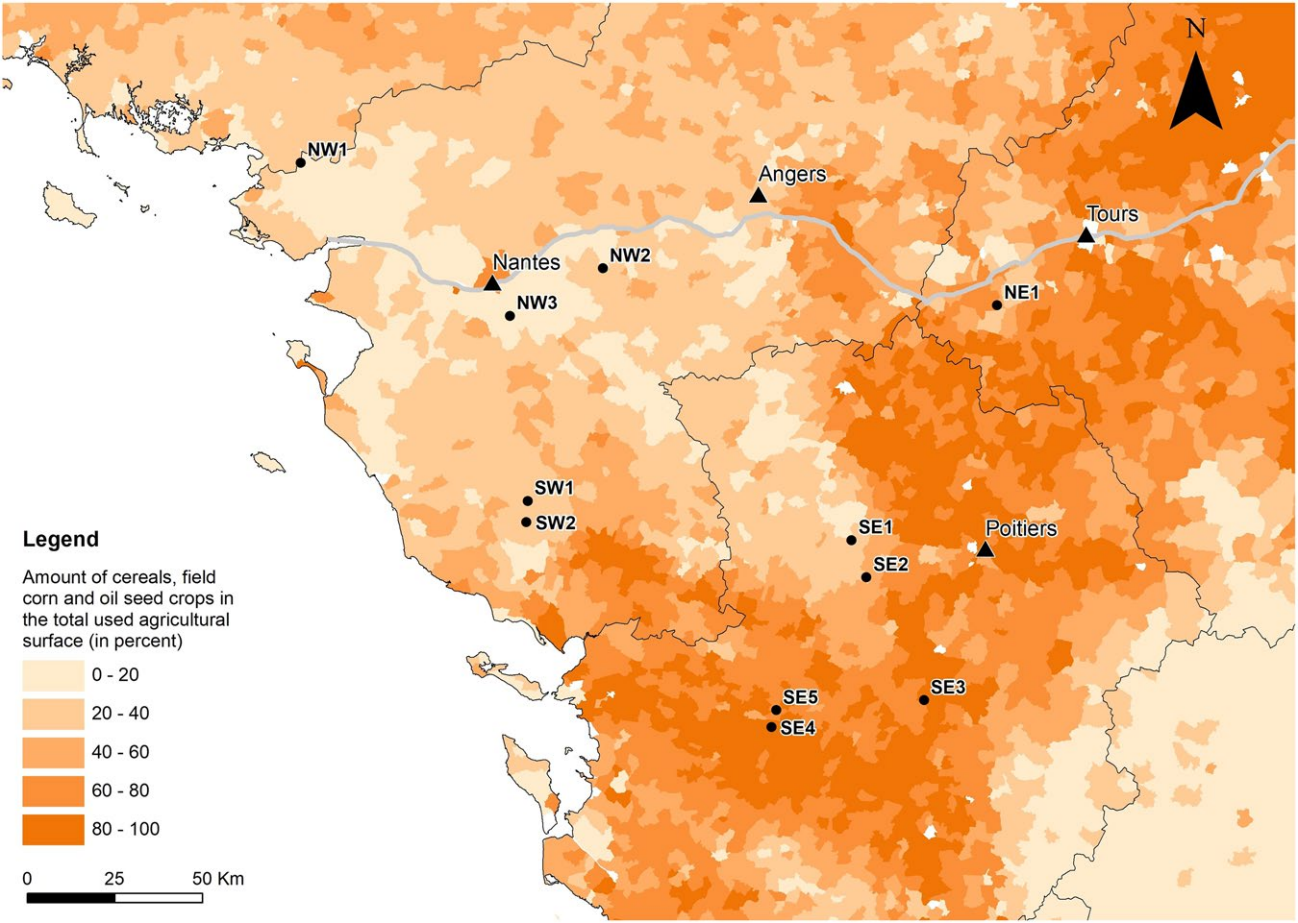
Amount (in percent) of large field crops (cereals, field corn and oil seed) in the total cultivated surface for the sampled area. Darker surfaces represent higer amounts of large field crops, the black dots show the location of the sites, the black triangles show the major citites in the area, the black lines represent region borders and the grey line is the Loire River. Data is from Agreste^75^. The figure was created using ArcMap 10.4 (Esri, Redlands, CA, USA, http://www.esri.com/arcgis/about-arcgis).

The presence of genetic structure between SE3 and the cluster including SE4 + SE5, situated 43 km away from each other, is in contradiction with the Mantel correlogram that showed a significant positive relationship between genetic and geographic distance up to 62 km (Fig. 4). This genetic structure could be explained by the agricultural landscape in the southern part of the southeastern group (SE3, SE4 and SE5) that is mostly composed of arable land used for intensive agriculture (Fig. 6). This type of landscape could have a detrimental influence on newt dispersal (Fig. 5) as cultivated areas and intensive pasture are generally avoided by amphibians^30^. Boissinot^31^ found that the presence of cultivated fields around a pond negatively influenced the probability of marbled newt presence in the pond. This is also supported by Trochet *et al*.^32^ who found longer travel distances for the marbled newt in forest compared to agricultural land. Our results also suggest that agricultural landscapes seem to increase population structure for the marbled newt. Indeed, we observed that when the proportion of arable land together with the distance between sites were increasing, pairwise F_st_ was also significantly increasing (Fig. 5). When only pairwise distance was taken into account to explain the variation of F_st_ in our sampling area, the model did not perform as well as when the interaction, proportion of arable land and distance between sites, was included (ΔAICc = 5.42). This result supports the hypothesis that agricultural landscape could be a barrier to marbled newt dispersal and could increase isolation between sites.

Another potentially intriguing result was that STRUCTURE did not detect genetic structure between NW1, NW2 and NW3, even though 91 km separated NW1 from NW2 (Fig. 1). Similarly, the pairwise F_st_ result between NE1 and NW2, separated by a distance of 114 km, was not significantly different from zero (Table 2) and was in contradiction with the overall trend displayed by the Mantel correlogram (Fig. 4). F_st_ for NW1 was not computed as too few individuals could be sampled at this locality (n < 20). However, the results of STRUCTURE and the DAPC show that NW1 belongs to the same cluster as NW2 and NW3. Beyond the relatively important distance separating NW1 from the other two sites, the probable connectivity between those localities is interesting due to the presence of potential barriers to dispersal between those sites. A large urbanised area (the city of Nantes) and a large river (the Loire River) (Fig. 1) might both decrease gene flow between the sampling site to the north (NW1) and sampling sites to the south (NW2 and NW3). Indeed, several studies on amphibians have found that urban areas and rivers could act as barriers and thereby increase population structure^33–36^. However, in our case, other unsampled areas around the city of Nantes could play the roles of stepping stones and/or reservoirs between the sampling sites. The local agricultural landscape might also play a positive role on the dispersal of individuals, as it features hedgerows, meadows and forest, the habitats favoured by marbled newts for efficient dispersal^37^, and a low amount of large field crop (Fig. 6). Regarding the Loire River, our results seem to be consistent with the studies of Gascon *et al*.^38^, Lougheed *et al*.^39^ and Johanet^40^, which found no significant barrier effect of rivers on gene flow. However, according to our data and the position along the Loire River of our sampling sites, we argue that the river might act as a corridor helping marbled newts dispersal. The Loire River can be relatively narrow during the dry season, with dense vegetation on the water and low stream flow, all of which facilitate the crossing of newts in summer^40^. Dispersal around the river could also occur during flood periods. In ponds bordering the river, we can also assume that larvae and eggs, which are often attached to vegetation, could be accidentally carried away by strong currents during the flood season^40–42^. Hence, there are good reason to believe the Loire River might not be a barrier to dispersal for the marbled newt and could even positively influence dispersal along its flow.

Overall, the observed connectivity supported by the lack of genetic structure at local scales (<15 km) in this study contrasts with the low migration and dispersal capacity of most amphibians^43^, and of the marbled newt in particular^20,44^. For the marbled newt, the maximum observed migration distance was 146 m for Jehle and Arntzen^44^ and 473 m for Trochet *et al*.^32^. This low migration capacity is generally explained by the dependency of amphibians on wetland areas^45^, as well as their slow terrestrial movements^46^. For a large newt species like the alpine newt, the maximum recorded dispersal distance was between 200 m^47^ and 500 m^48^. Langton *et al*.^49^ observed a maximal dispersal distance of approximately 1000 m around the reproduction pond for another large newt species, the crested newt. Regarding genetics, our results are in accordance with Smith and Green^50^. According to their meta-analysis, in general, anurans and salamanders do not present any structure for distances lower than 10 km. This is also consistent with the results of Isselin-Nondedeu *et al*.^22^ who found, based on genetic analysis, that the small palmate newt *Lissotriton helveticus* was able to rapidly colonise newly restored ponds within a forest.

Our results indicate that the survival and dispersal of marbled newt populations could be largely dependent on landscape type and the associated dispersal corridors available in the surrounding areas. Our study supports the idea that populations far apart might be genetically well connected, provided that good dispersal paths such as rivers^51^, hedgerows and forests exist. Such connection might facilitate movements and dispersal between reproduction sites, while forests are also used as a shelter against predation and desiccation^17^. Because of the low dispersal capacity of the marbled newt, a dense pond network could be needed to maintain genetic exchange at distances >10 km in areas with a large amount of arable land. Moreover, large rivers and cities did not seem to represent barriers to dispersal in our study. In fact, the Loire River seems to act as a dispersal corridor for the marbled newt and possibly also for other amphibians. A test to identify the direction of gene flow between our localities could provide an improved understanding of the influence of the river on newt dispersal. On the other hand, genetic structure was found in areas with large amounts of arable land. Therefore, presence of arable land seems to have a greater impact on the marbled newt’s gene flow than do large anthropic constructions, such as cities. In light of our results and of current habitat losses in the west of France, we recommend the following guidelines for the conservation of marbled newt populations: (1) to maintain a dense network of reproduction sites (suitable ponds in proximity of forested areas); (2) to protect hedgerow, meadow and forest landscapes and focus on the restoration of natural areas where intensive agricultural landscapes predominate; (3) finally, it could be beneficial to protect ponds close to rivers in order to promote long distance gene flow.

## Material and Methods

### Study sites and sampling

Samples were collected on 11 different locations in 34 different ponds distributed at a regional scale in Western France (see Fig. 1). The study areas were selected to provide a diversity of agricultural landscapes (conserved meadows or large field crops) and a variety of situations regarding the presence or absence of nearby forest. Our sampling design provided sites separated by three different levels of geographical distances: inter-region (>100 km), intra-region (between 100 km and 10 km) and local (<10 km). This design allowed us to compare genetic diversity and population structure at different spatial scales. Ponds in an area <4 km^2^ and unseparated by fragmenting elements were considered as one location in the analysis in order to have a large enough sample size. This threshold was chosen to be more conservative than Smith and Green^50^, who estimated that population differentiation for amphibians mostly occurs at distances above 10 km.

A total of 334 individuals were sampled for the analysis. Sampling sites were located in different geographical regions. The northern sampling sites (NW1, NW2, NW3 and NE1) were along the Loire Valley. The Loire Valley is dominated by a mix of large and small pastures delimited by hedgerows called “bocage”. This landscape is also characterised by a low proportion of large field crops (arable land): cereals, field corn and oil seed (as defined in Laurent^52^) (Fig. 6). An exception was site NE1, where the ponds were situated in a forest-dominated landscape. There was a gap of more than 100 km in a straight line without sampling sites between the northwestern sampling sites (NW1, NW2, NW3) and the northeastern sampling site NE1 (Fig. 1). Between the northern and southeastern sampling sites, the landscape was dominated by extensive farming with a weak density of hedgerows and few groves. In the southwest (SW1 and SW2), the density of bocage was high around the sampling sites. Between southwestern (SW1 and SW2) and southeastern (SE1, SE2, SE3, SE4, SE5) sampling groups (Fig. 1), there was extensive farming with large field crops (cereals, corn and oil seed) (Fig. 6) with a low density of hedgerows and few groves. In the southeastern group, SE1 and SE2 were largely dominated by bocage whereas SE3, SE4 and SE5 were surrounded by landscape largely dominated by intensive farming. SE4 and SE5 were in an isolated forest and SE3 was in a small isolated bocage.

### Ethical note

All trapping and handling procedures were in accordance with the relevant guidelines and regulations and were approved by the appropriate authority, the Directions Régionales de l’Environnement, de l′Aménagement et du Logement (DREAL). Only non-invasive methods were used to collect DNA and individuals were released immediately after sampling^53^. Moreover, chytridiomycosis protocols were followed as advised by the French herpetological society (Société Herpétologique de France). No individuals were injured during capture and handling and all were successfully released after DNA sampling.

### DNA sampling and genetic analysis

DNA collection of epithelial cells was realised by buccal swab following Pidancier *et al*.^54^. For each pond all captured individuals were sampled at once during the same session in order to avoid replication. Individuals were caught either with landing nets or Ortmann’s funnel traps^55^.

DNA extractions were performed using the salting out protocol of Sunnucks and Hales^56^. To compare population structures, 10 microsatellite markers were used in this study. Nine were developed specifically for the marbled newt^24^ and one (Tcri27) was developed for the crested newt^57^. PCR and genotyping were performed by the Gentyane INRA platform (Clermont-Ferrand, France). We analysed the genotyped data with Genemapper v4.0 (AppliedBiosystems™).

### Data treatment

Deviation from Hardy-Weinberg equilibrium was calculated using GENEPOP v4.2^58^ and significance levels were adjusted with Bonferroni correction. A possible Wahlund effect for populations presenting a deficit in heterozygotes was tested for the correlation between F_is_ and F_st_ per locus. F_is_ and F_st_ per locus were obtained with the R package hierfstat v0.04-22^59^ implemented in R v3.3.2^60^, then a linear regression was calculated in order to observe the relationship between both variables^25^. Expected heterozygosity (H_epx._), observed heterozygosity (H_obs._), and average number of alleles per locus were calculated with the software Genetix v4.05.2^61^. The allelic richness (Ar.) was plotted using allele rarefaction curves, as calculated by the package PopGenKit v1.0 (R package, R Core Team), implemented in R v3.2.2^62^. After visual verification, the minimum sample size was set to 14 in order to have a good estimation of the number of alleles. Pairwise F_st_ and significance probabilities were obtained with the software Arlequin (Ver 3.5)^63^. Only sample sites (n = 8) with more than 20 individuals were kept for this analysis. This minimum was set according to Kalinowski^64^, as it give good results for F_st_ larger than 0.01. F_st_ significance was corrected for multiple testing with the Bonferroni method.

Bayesian estimations of population structure were inferred with the software STRUCTURE (2.3.4)^26^ using a burn-in period of 150,000 followed by 10^6^ Markov chain Monte Carlo (MCMC) iterations. Default settings were used (Admixture model) for all parameters but for the LOCPRIOR option (the sample location was used as a prior), which was enabled as it tends to perform better than models without LOCPRIOR enabled^65^. For each analysed dataset, 10 replicate runs were performed for each value of the number of clusters (K) from 1 to 10. The most likely number of clusters (K) was determined using the calculation of delta K (ΔK) described in Evanno *et al*.^66^, as implemented by the web interface of STRUCTURE HARVESTER^67^, but also visually directly from STRUCTURE’s bar plots (Fig. 2). Sampling sites were assigned to a specific population according to the highest average membership coefficient. For each cluster detected by STRUCTURE, analysis was run again with the same parameters in order to detect more subtle substructure^68^ (Fig. 2b,c). When the STRUCTURE bar plot showed that individuals could not be assigned to different clusters and the ΔK was low, the dataset was considered as one cluster.

Discriminant analysis of principal components (DAPC) was run using the dapc function in the R package adegenet^69^, as a complementary analysis for population structure. This method applies a discriminant analysis on data previously transformed using a principal component analysis (PCA). DAPC optimises the genetic variance between groups while minimising the variance within groups, in order to show better separation of the different groups. For the PCA calculation, after visual interpretation, we decided to retain 35 PCs as it conserved 96% of the variance. Regarding the discriminant analysis, the first two eigenvalues were kept, after visual interpretation, as they were carrying most of the information. The results of the DAPC were gathered in a scatterplot, with individuals represented as dots and the different groups as inertia ellipses.

The smallest clusters detected by STRUCTURE were used as predefined groups for the AMOVA in order to detail our population structure. The AMOVA was calculated with the software Arlequin (Ver 3.5)^63^.

Isolation by distance among populations was tested with a Mantel correlogram using the matrix of pairwise Euclidian distance (in kilometres) between sample sites and the matrix of pairwise standardised genetic distances between sampling sites (F_st_/1 − F_st_). The calculation was performed with the R package vegan (vegan v 2.3–3 R package, R Core Team), function mantel.correlog, with 9999 permutations and significance level <0.05 (adjusted for multiple testing with the Holm methods).

### Landscape analysis

We tested the influence of the agricultural landscape on genetic distance by analysing habitat types between sampling sites. To do so we created, on Arcgis 10.4 (Esri, Redlands, CA, USA, http://www.esri.com/arcgis/about-arcgis), a 10 km wide corridor between every sampling site. We then used the CORINE (Coordination of Information on the Environment) land cover data from Copernicus land cover monitoring services^70^ to record the different habitat types within each corridor. The amount (in percent) of different habitat types within each corridor was then calculated with the software FRAGSTATS (v4.2)^71^. In order to detect the influence of large field crops on genetic distance we used the proportion of arable land (code 21) within each corridor and accounted for the distance between sites. We chose arable land as it is the CORINE land cover habitat that best represents the large field crops and intensive agricultural activities. In our sampling area we only had one type of arable land, non-irrigated arable land (code 211), and so for the rest of the article we will refer to it simply as arable land. We then created a linear model with pairwise F_st_ as a response variable and proportion (in percent) of arable land, and pairwise distance as explanatory variables. We also included the interaction between the two explanatory variables in order to account for the combined effect of both pairwise distance and amount of arable land. The three candidate models were then compared using Akaike’s Information Criterion corrected for sample size (AIC_c_)^72^ with the function model.sel from the R package MuMIn^73^. The model with the lowest AIC_c_ score was selected, and if ΔAICc was <4, the most parsimonious model was chosen. Finally, parameters that had zero in their 95% confidence interval were considered non-informative^27^. A graph presenting the results of the best model was then created using the R package ggplot2^74^ (Fig. 5).

## Data Availability

The datasets generated and analysed during the current study are available from the corresponding author on reasonable request.
