# Author Correction: Agricultural landscapes and the Loire River influence the genetic structure of the marbled newt in Western France

**DOI:** 10.1038/s41598-018-35692-x

**Published:** 2018-11-19

**Authors:** Jean-Marc Costanzi, Pascal Mège, Alexandre Boissinot, Francis Isselin-Nondedeu, Sandra Guérin, Olivier Lourdais, Audrey Trochet, Quentin Le Petitcorps, Agathe Legrand, François Varenne, Pierre Grillet, Sophie Morin-Pinaud, Damien Picard

**Affiliations:** 1 Faculty of Technology, Natural Sciences and Maritime Sciences, University of South-Eastern Norway, Gullbringvegen 36, Bø i Telemark, 3800 Norway; 2 Département de Biologie, UFR de Sciences, Université d’Angers, 2 Boulevard de Lavoisier, 49000 Angers, France; 3 Centre d’Études Biologiques de Chizé, CNRS et Université de la Rochelle – UMR 7372, F-79360 Villiers en Bois, France; 4 Ecole Polytechnique de l’Université François Rabelais, Département d’Aménagement et d’Environnement, UMR 7324 – CNRS CITERES 33-35 allée Ferdinand de Lesseps, 37200 Tours, France; 5 IMBE, UMR Université Aix-Marseille Avignon, 7263-CNRS, 237-IRD IRPNC (Ingénierie de la Restauration des Patrimoines Naturels et Culturels), Avignon, France; 6 EPHE, PSL Research University, CNRS, UM, SupAgro, IRD, INRA, UMR 5175 CEFE, F-34293 Montpellier, France; 7 CNRS, ENFA, UMR5174 EDB (Laboratoire Evolution et Diversité Biologique), Université Paul Sabatier, 118 route de Narbonne, Toulouse, F-31062 France; 8 Station d’Ecologie Théorique et Expérimentale, UMR 5321, Moulis, F-09200 France; 9 LPO Vendée - La Brétinière, 85000 La Roche-sur-Yon, France; 10 10 rue de la Sayette, 79340 Vasles, France; 11 Office National de la Chasse et de la Faune Sauvage - Unité Avifaune migratrice, Direction de la Recherche et de l’Expertise, F-79360 Villiers en Bois, France

Correction to: *Scientific Reports* 10.1038/s41598-018-32514-y, published online 21 September 2018

The original version of this Article contained errors in the spelling of the authors Jean-Marc Costanzi, Pascal Mège, Alexandre Boissinot, Francis Isselin-Nondedeu, Sandra Guérin, Olivier Lourdais, Audrey Trochet, Quentin Le Petitcorps, Agathe Legrand, François Varenne, Pierre Grillet, Sophie Morin-Pinaud & Damien Picard, which were incorrectly given as Costanzi Jean-Marc, Mège Pascal, Boissinot Alexandre, Isselin-Nondedeu Francis, Guérin Sandra, Lourdais Olivier, Trochet Audrey, Le Petitcorps Quentin, Legrand Agathe, Varenne François, Grillet Pierre, Morin-Pinaud Sophie & Picard Damien respectively.

In addition there was an error in Affiliation 5, which was incorrectly given as ‘IMBE, UMR Université Aix-Marseille Avignon, 7223-CNRS, 237-IRD IRPNC (Ingénierie de la Restauration des Patrimoines Naturels et Culturels), Aix-en-Provence, France’. The correct affiliation is listed below:

IMBE, UMR Université Aix-Marseille Avignon, 7263-CNRS, 237-IRD IRPNC (Ingénierie de la Restauration des Patrimoines Naturels et Culturels), Avignon, France.

These errors have now been corrected in the HTML and PDF versions of the Article.

